# Prevalence of carbapenem-resistant Enterobacteriaceae and emergence of high rectal colonization rates of *bla*_OXA-181_-positive isolates in patients admitted to two major hospital intensive care units in Kuwait

**DOI:** 10.1371/journal.pone.0241971

**Published:** 2020-11-17

**Authors:** Amani H. Al Fadhli, Wafaa Y. Jamal, Vincent O. Rotimi

**Affiliations:** Department of Microbiology, Faculty of Medicine, Kuwait University, Kuwait City, Kuwait; University of Georgia, UNITED STATES

## Abstract

**Background:**

Fecal colonization by carbapenem-resistant Enterobacteriaceae (CRE) can be the main reservoir for transmission of these resistant organisms especially in the Intensive Care Units (ICUs).

**Aim:**

This study was conducted to evaluate the rate of rectal carriage and molecular characterization of CRE in patients hospitalized in the ICUs of 2 major hospitals (Adan and Mubarak Al Kabeer Hospitals) in Kuwait.

**Materials and methods:**

Rectal swabs were collected from all patients at admission, 48 h after admission and once weekly from April 2017- March 2018. Initial CRE screening was carried out on MacConkey agar on which meropenem disc 10μg was placed. Identification of isolates was by API 20E. Susceptibility testing was performed using the E-test method. Polymerase chain reaction (PCR) was used to detect the carbapenemase-encoding genes. Clonal relationship was investigated by pulsed-field electrophoresis (PFGE). Genes of *bla*_OXA-181_ and *bla*_NDM-5_–carrying plasmids were detected in some strains.

**Results:**

A total of 590 patients were recruited into the study. Of these, 58 were positive for CRE, giving a prevalence of 9.8%; 25/320 (7.8%) in Adan and 33/270 (12.2%) in Mubarak Al Kabeer Hospitals. All isolates were resistant to multiple antibiotics. Resistance rates to colistin and tigecycline were 17% and 83%, respectively. Single genes of *bla*_OXA-181_ were detected in isolates from 38 (65.5%) out of 58 patients and in 5 patients colonized by *bla*_OXA-48_-positive CRE. A combination of 2 genes was detected in 12 isolates; 5 *bla*_KPC-2_ and *bla*_OXA-181_, 4 *bla*_VIM-1_ and *bla*_OXA-181_, and 3 *bla*_NDM-5_ and *bla*_OXA-181_. PFGE showed an overall level of similarity of 38%. Southern hybridization studies localized the *bla*_OXA-181_ and *bla*_NDM-5_ genes to a large plasmid of 200kb in 3 *K*. *pneumoniae* isolates and a small plasmid of 80kb in 2 *E*. *coli* isolates, respectively.

**Conclusion:**

The prevalence of CRE colonization in the 2 hospital ICUs was relatively high and the emergence of *bla*_OXA-181_-mediated CRE is a cause for concern as there is the possibility of rapid horizontal spread among hospital patients in Kuwait. Active surveillance of CRE in the ICUs is highly recommended to stem its spread.

## Introduction

Gut flora is a major reservoir for Enterobacteriaceae which are potentially pathogenic to hospitalized patients especially those in the Intensive Care Units (ICUs). These Enterobacteriaceae have acquired resistance to several antimicrobial agents over time including the cephalosporins due inactivation by extended-spectrum β-lactamases (ESBLs) and AmpC production [1, 2]. This has led to excessive use of carbapenems for treating documented infections as well as for empirical therapy of suspected ESBL-associated infections especially those acquired in the ICUs [3]. The consequence of this has been accumulation of selective pressure for carbapenem resistance and the emergence of carbapenem-resistant Enterobacteriaceae (CRE). CRE infections are among the most problematic clinical challenges due to limited therapeutic options, limited isolation facilities, and higher financial cost [4]. They are increasingly reported worldwide primarily due to cumulative awareness globally and better identification methods in the hospital diagnostic laboratories.

Fecal colonization by CRE has been rarely investigated compared with colonization by ESBLs-producing Enterobacteriaceae in non-outbreak settings. In Kuwait, the emergence of carbapenem resistance among members of the family Enterobacteriaceae was not reported until 2012 when 2 patients infected with nosocomial acquired NDM-1 producing *Klebsiella pneumoniae* were encountered in the adult ICU of Mubarak Al Kabeer hospital [5]. Since then more and more infected or colonized patients by CRE have been encountered in many hospitals in Kuwait in the subsequent years [6–8]. The isolates responsible for these infections/colonizations have been NDM-1, VIM-4, and OXA-48-producing strains. OXA-181-producing CRE have never been isolated before as clinical or colonizing strains anywhere in Kuwait. The enzyme is a variant of OXA-48 by 4 amino acid substitutions at the Thr104Ala, Asn110Asp, Glu175Gln, and Ser179Ala positions [9]. It was initially identified in *Enterobacter cloacae* and *K*. *pneumoniae* strains isolated from patients in India in 2007 [10]. Since then OXA-181-producing Enterobacteriaceae has been reported in many other countries around the world including Nepal, Sri Lanka, Bangladesh, Canada, France, The Netherlands, Oman, Romania, South Africa, and United Kingdom [11–13]. Controlling the spread of CRE isolates involves reduction in antibiotic use, adherence to the infection control guidelines, and early detection of rectal colonization. Early detection of carriers or colonizers allows for early and rapid establishment of contact precautions to prevent CRE transmission to other patients. Infection with CRE has important implications on the care of the patients especially those in the ICUs because of limited therapeutic options. There is also a risk of outbreaks which may be attributed to environmental contamination or break in the infection control guidelines [14, 15]. This study was designed to evaluate the rectal prevalence of and molecular characteristics of CRE among patients admitted to two adult ICUs in Adan and Mubarak Al Kabeer Hospitals.

## Materials and methods

*Study design*: This study was conducted for a period of one year (April 2017 to March 2018) in the 32–bedded and 26-bedded medical/surgical ICUs of Adan and Mubarak Al Kabeer Hospitals, Kuwait, respectively. These hospitals are large University-affiliated secondary and tertiary hospitals with 935 and 830 beds, respectively. They are located about 16 km apart responsible for a catchment population of about 2.5 million people.

### Patients

A total of 590 consecutive patients admitted to the ICUs of Adan Hospital (n = 320; 54.2%) and Mubarak Al Kabeer Hospital (n = 270; 45.8%) over a period of 12 months were recruited into this study. Rectal swabs were collected from these patients on the day of admission, after 48 hours, and then at weekly intervals, till the day of discharge or death. A colonized patient with CRE on admission was defined as a carrier from the community. Nosocomial ICU-acquired CRE isolates were defined as those acquired after 48 hours of admission. The acquisition rate was determined by follow-up rectal cultures obtained from those negative at admission every week until the patient was discharged or deceased. The definition of acquisition rate was the number of patients who were not carriers on admission but became carriers later, divided by the number of patients who are not carriers on admission [16]. Demographic bio-data, e.g. age, gender, sample type, nationality, travel history, previous hospitalization within one year of current admission, diagnosis, co-morbidity, and prior antibiotic use, was recorded.

### Ethics statement

Institutional ethical approval was obtained from the authorizing Medical Ethics Committee of the Ministry of Health (permit number 2016/272). Collection of the specimens was conducted according to the Declaration of Helsinki and with particular institutional ethical and professional standards. Written informed consent was obtained from the patients or their guardians and no additional clinical specimens were collected for the purpose of the study.

### Microbiological studies

Rectal swabs were inoculated directly onto MacConkey (MAC) agar (Oxoid, Basingstoke, Hants, UK) on which 10μg meropenem disk (Becton Dickinson Ltd., Research Park Drive, North Ryde, Australia) was placed which allowed the establishment of optimal zone diameters for the screening of carbapenem-resistant Gram-negative bacteria. After incubation for 24 h at 37°C in an incubator (GallenKamp Economy Incubator size 2, Akribis Scientific Limited, Common Farm, Frog Lane, Pickmere, Knutsford, Cheshire), a reduced zone of inhibition of ≤19 mm around the meropenem disc was considered a potential CRE [17–19]. After the growth on MAC, identification of the isolates to the species level was done using the API 20E (bioMérieux, Marcy, L’Etoile, France).

### Antibiotic susceptibility testing

The minimum inhibitory concentrations (MICs) of the antibiotics tested were determined by using E-test (bioMérieux) according to the manufacturer’s instruction. Antibiotics tested were the following: amikacin, cefoxitin, ciprofloxacin, colistin, ertapenem, imipenem, meropenem, and tigecycline. Interpretation of the results was carried out according to the Clinical and Laboratory Standards Institute [17] interpretative criteria. Tigecycline and colistin susceptibility were interpreted according to the EUCAST guidelines [19]. The following control strains, *Escherichia coli* ATCC 25922, *Pseudomonas aeruginosa* ATCC 27853, and *E*. *coli* ATCC 35218 were included in each run. To compare the susceptibilities of different groups, the MICs that inhibited 90% (MIC_90_) and 50% (MIC_50_) of the isolates were calculated. CRE was defined as an Enterobacteriaceae isolate that showed decreased susceptibility to ertapenem (MIC>0.5μl/ml), imipenem (MIC>1μl/ml), and/or meropenem (MIC>1μl/ml) regardless of carbapenemase production [17].

### Phenotypic and molecular characterization

The phenotypic production of carbapenemase by the isolates was investigated by using PCR and sequencing. PCR assays were done using previously described primers designed to detect Ambler class A (*bla*_KPC_), class B (*bla*_VIM_, *bla*_IMP_, *bla*_NDM_) and class D (*bla*_OXA-48_ and *bla*_OXA-like_) carbapenemase genes [20–22]. Sequencing was performed using the GeneAmp PCR system 9700 by cycle sequencing with BigDye termination (AB Applied Biosystems, Foster City, California, USA). The sequencing results were analyzed using software from the National Center for Biotechnology Information (www.ncbi.nlm.nih.gov).

### Pulsed-field gel electrophoresis (PFGE)

Clonality of the CRE isolates was investigated by macrorestriction analysis of the genomic DNA with *Xba*I (New England Biolabs, Ipswich, MA, USA). The genomic DNA fragments were separated by PFGE in the CHEF-DR III system (Bio-Rad, Hemel Hempstead, UK). Electrophoresis conditions were pulsed-times ranging from 5 to 45 sec for 20 h at 6 V/cm at 14°C. Restriction patterns were analyzed according to previously described criteria [23]. In the PFGE analysis, the isolates were considered as highly related (100–97.5%), related (97.5–80%) and unrelated (<80%).

### Plasmid analysis

Plasmids carrying *bla*_OXA-181_ and *bla*_NDM-5_ were detected and characterized in 3 *K*. *pneumoniae* (MK-18, MK-19, Adan-42) and 2 *E*. *coli* (MK-3, MK-6,) isolates by alkaline lysis method using episomes in *E*. *coli* 39R861 and *E*. *coli* V517 as molecular mass controls according to the previously described method of Kado and Liu [24]. It was followed by hybridization with *bla*_OXA-181_ and *bla*_NDM-5_ probes, using the ECL Random-Prime Labelling and detection system (Amersham Pharmacia Biotech, Little Chalfont, UK). The NDM-5 containing plasmids were conjugally transferred into *E*. *coli* J53RAZ [25].

### Statistical analysis

The Epical 2000, version 1.02 (Brixton Health, Llanidloes, Powys, Wales, UK) was used to compare 2 proportions—percentages with 95% confidence interval and one-sided *P*-value. A Pearson’s Chi-squared test (IBM SPSS Statistics 26) was carried out to assess whether nationality and comorbidity were related significantly. *P* value of ≤0.05 was considered as statistically significant.

## Results

### Demographic characteristics

**Fig 1:**
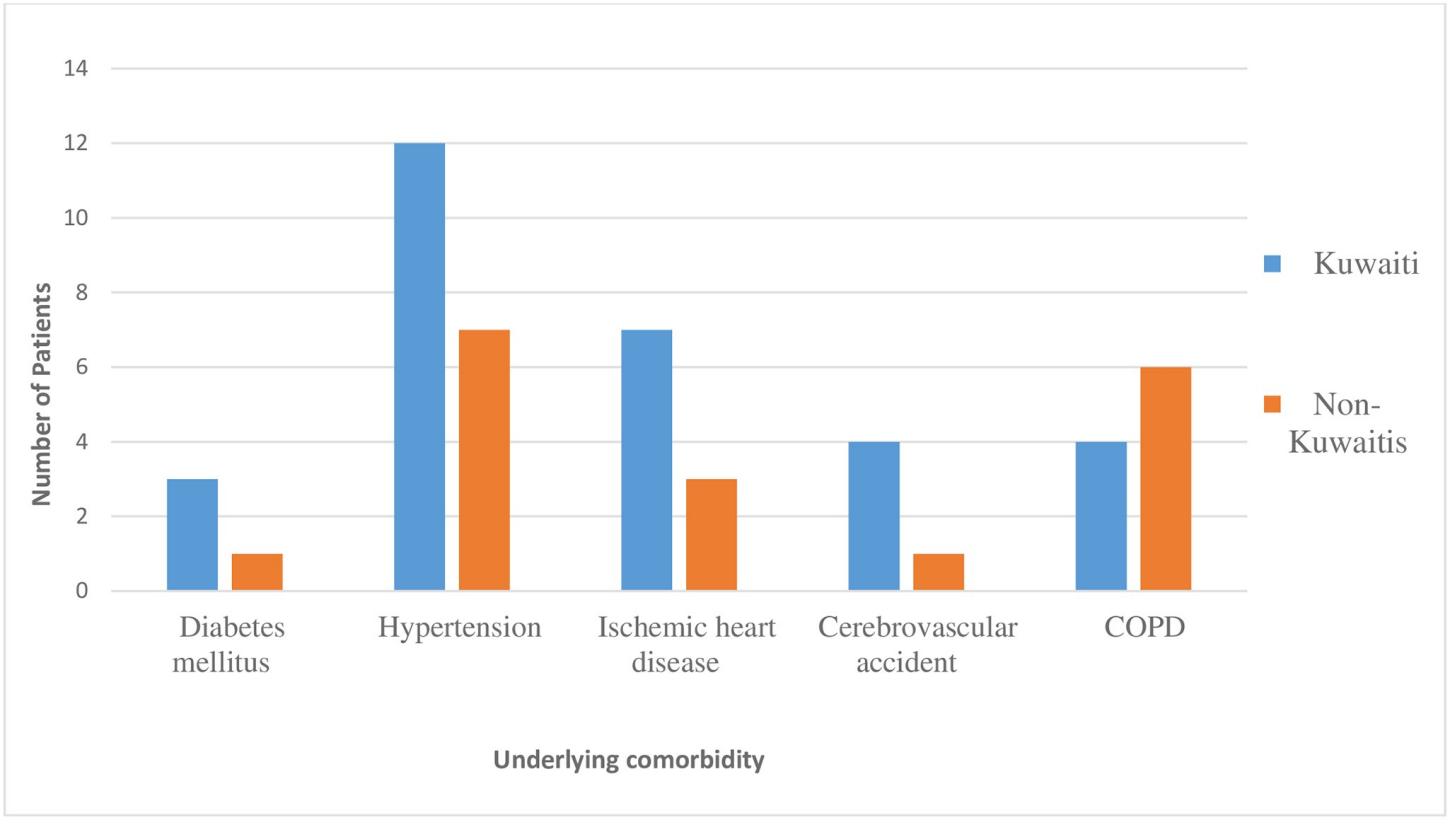
Underlying comorbidity of patients colonized with CRE and their nationality.

**Fig 2:**
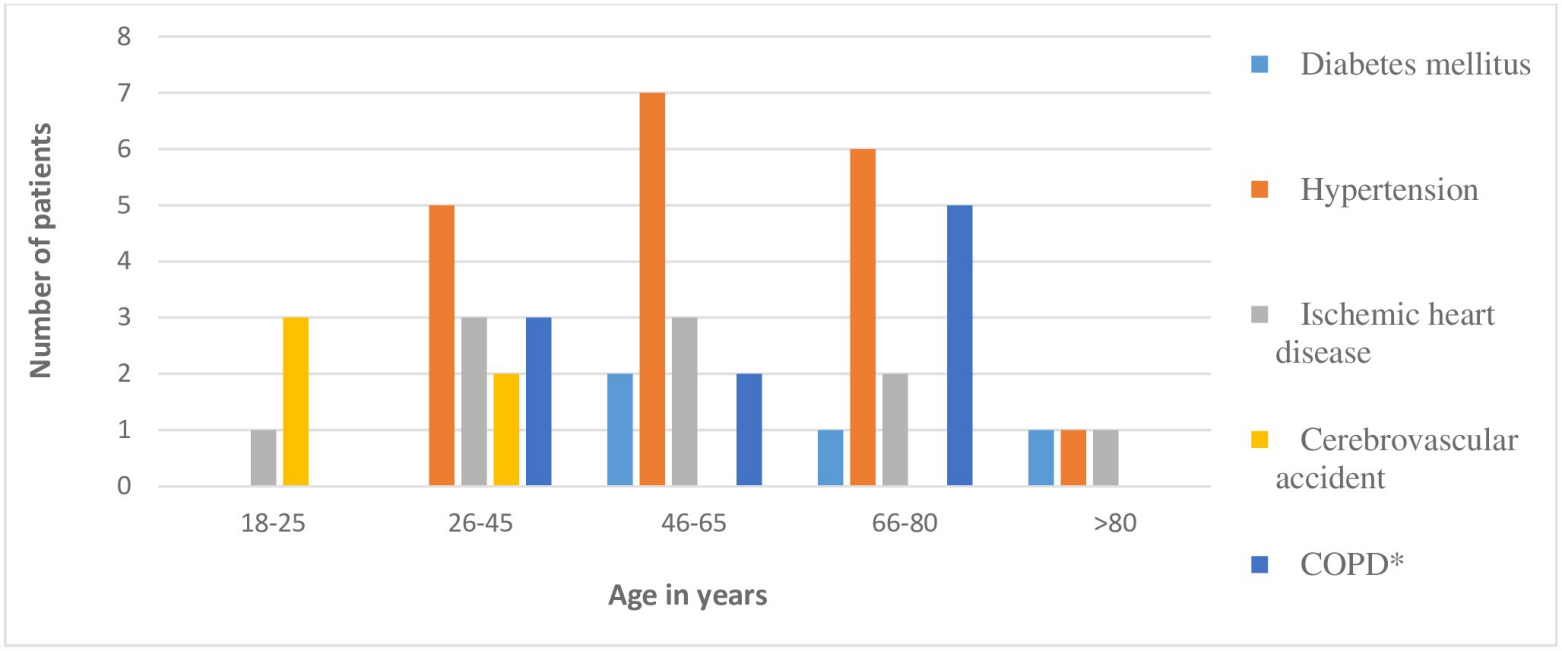
Underlying comorbidity of patients colonized with CRE and their age group.

The colonization rates of the ICU patients during the study period were 7.8% (25 / 320) at Adan Hospital, and 12.2% (33/270) at Mubarak Al Kabeer Hospital. The demographic characteristics of all CRE-positive patients were generally comparable and they are summarized in Table 1. None of the general characteristics of the patients attained statistical significance between the two hospitals, except chronic obstructive pulmonary disease (COPD) comorbidity (*P* = 0.007). Of the 58 colonized patients, 33 (56.9%) were males and 25 (43.1%) females, with a male-to-female ratio of 1:0.8. The ages ranged from 18 to 89 years (mean = 50.4 years). Approximately 70% of the patients were over the age of 45 years in both hospitals; 20/25 (80%) in Adan versus 23/33 (69.7%) Mubarak Al Kabeer, (*P*>0.05). Nine (36%) versus 12 (36.4%), respectively were in the age group 46–65 and 10 (40%) versus 10 (30.3%), respectively in the 66–80 age group, (*P*>0.05). Thirty-nine (67.2%) were Kuwaitis, followed by 10 (17.2%) Indians and seven (12.1%) Egyptians. Intra-hospital, but not inter-hospital, differences between Kuwaitis and non-Kuwaitis was statistically significant (*P*<0.0001) in both hospitals. The most prominent comorbidities were hypertension (19/58: 32.7%), ischemic heart diseases (10/58: 17.2%), and COPD (10/58: 17.2%); none of these, except COPD (*P*<007), attained inter-hospital statistical significance. Other underlying conditions included cerebrovascular accident (5/58: 8.6%) and diabetes mellitus (4/58: 6.9%). A total of 22 (37.9%) gave history of previous treatment with a carbapenem and 10 (17.2%) with intravenous cephalosporins prior to admission into the ICU. Figs 1 and 2 show the inter-connecting nationality group and age group with underlying conditions.

**Table 1:** General characteristics of the intensive care unit patients colonized by carbapenem–resistant Enterobacteriaceae.

Demographic variables		No (%) of patients colonized in		*P* value
	Total no of patients (%)	Adan hospital (N = 25)	Mubarak hospital (N = 33)	
*Age group in years*				
18–25	3 (5.2)	1 (4)	2 (6.1)	0.8
26–45	12 (20.7)	4 (16)	8 (24.2)	0.6
46–65	21 (36.2)	9 (36)	12 (36.4)	0.8
66–80	20 (34.5)	10 (40)	10 (30.3)	0.6
>80	2 (3.4)	1 (4)	1 (3)	0.5
*Sex*				
Male	33 (56.9)	14 (56)	19 (57.6)	0.9
Female	25 (43.1)	11 (44)	14 (42.4)	0.9
*Nationality*				
Kuwaiti	39 (67.2)	19 (76)	20 (60.6)	0.3
Non-Kuwaitis	19 (32.7)	6 (24)	13 (39.4)	0.6
*Underlying conditions*				
Diabetes mellitus	4 (6.9)	3 (12)	1 (3)	0.4
Hypertension	19 (32.7)	8 (32)	11 (33.3)	0.9
Ischemic heart disease	10 (17.2)	2 (8)	8 (24.2)	0.2
Cerebrovascular accident	5 (8.6)	2 (8)	3 (9.1)	0.7
COPD***	10 (17.2)	0 (0)	10 (30.3)	0.007
*Previous hospitalization*				
Yes	6 (10.3)	2 (8)	4 (12.1)	0.9
No	52 (89.6)	23 (92)	29 (87.9)	0.9
*Previous/current antibiotic use*				
Carbapenems	22 (37.9)	10 (40)	12 (36.3)	0.9
Cephalosporins	10 (17.2)	5 (20)	5 (15.2)	0.9
Other β-lactams	2 (3.4)	0 (0)	2 (6)	0.6
Aminoglycoside	7 (12.1)	3 (12)	4 (12.1)	0.7
Fluoroquinolone	8 (13.8)	3 (12)	5 (15.2)	0.9
Macrolides	6 (10.3)	3 (12)	3 (9.1)	0.9
Polymyxin	3 (5.2)	1 (4)	2 (6.1)	0.8

*P<0.001

**P<0.0001

***COPD = chronic obstructive pulmonary disease. Difference between Adan and Mubarak hospitals was statistically significant *P* = 0.007.

### Prevalence of rectal colonization by CRE

Out of the 58 patients colonized by CRE in the two ICUs, 12 were colonized on the day of admission and 46 acquired theirs after 48 hours of admission, giving prevalence rates of 2% and 7.8% community-acquired and hospital-acquired CRE, respectively; this difference is statistically significant (*P*<0.00009). Analysis per hospital revealed 2.2% (7/320) community-acquired and 5.6% (18/320) hospital-acquired rates (*P*<0.041), respectively in Adan Hospital, and 1.9% (5/270) and 10.4% (28/270), respectively (*P*<0.00007) in Mubarak Al Kabeer Hospital. Of these colonized patients, 14 (24.1%) died during the study period: 7 each from Adan and Mubarak.

### CRE and antimicrobial susceptibility

The breakdown of the species of the CRE isolates is shown in Table 2. The most prevalent was *K*. *pneumoniae*, representing 29 (50%) of all isolates, followed by *E*. *coli*, 11 (19%), *Enterobacter* spp. 5 (8.6%), *Serratia* spp. 5 (8.6%), and others, 6 (13.7%).

**Table 2:** Distribution of carbapenem-resistant Enterobacteriaceae isolates in the two hospitals.

Bacteria	Number (%) of CRE isolates			
	Adan hospital (N = 25)		Mubarak hospital (N = 33)	
	Community acquired (N = 7)	Hospital acquired (N = 18)	Community acquired (N = 5)	Hospital acquired (N = 28)
*Escherichia coli*	0	1 (4)	1 (20)	9 (32.1)
*Klebsiella pneumoniae*	5 (71.4)	11 (64)	2 (40)	11 (39.3)
*Klebsiella oxytoca*	0	0 (0)	0	2 (7.1)
*Enterobacter cloacae*	0	1 (4)	1 (20)	2 (7.1)
*Enterobacter gergoviae*	0	0 (0)	0	1 (3.6)
*Morganella morganii*	1 (14.3)	1 (4)	0	0 (0)
*Pantoea agglomerans*	0	1 (4)	0	0 (0)
*Proteus mirabilis*	0	0 (0)	0	1 (3.6)
*Roseomonas gilardii*	1 (14.3)	1 (4)	0	0 (0)
*Salmonella enterica*	0	1 (4)	0	0 (0)
*Shigella boydii*	0	0 (0)	0	1 (3.6)
*Serratia odoriferae*	0	1 (4)	0	0 (0)
*Serratia marcescens*	0	0 (0)	1 (20)	0 (0)
*Serratia plymuthica*	0	1 (4)	0	1 (3.6)
*Serratia rubidaea*	0	1 (4)	0	0 (0)

The MIC range, MIC_50_, MIC_90_, and percentage of resistance of the CRE isolates are given in Table 3. All the *K*. *pneumoniae* isolates from Adan hospital were resistant to cefoxitin, ciprofloxacin, ertapenem, imipenem and meropenem. About 75% were resistant to tigecycline with MIC_50_ and MIC_90_ of 2 μg/ml and 8 μg/ml, respectively while 44% were resistant to colistin with MIC_50_ and MIC_90_ of 1 μg/ml and 8 μg/ml, respectively. In Mubarak Al Kabeer Hospital, *K*. *pneumoniae* exhibited high resistance to the antibiotics with MIC_90_s >256 μg/ml for amikacin and cefoxitin, and > 32μg/ml for ciprofloxacin, ertapenem, imipenem, and meropenem.

**Table 3:** Minimum inhibitory concentrations (MICs) of the antimicrobial agents tested against carbapenem-resistant Enterobacteriaceae (CRE) isolates.

Bacteria/antibiotics (breakpoint)	Susceptibility of the CRE isolates per hospital							
	Adan hospital				Mubarak hospital			
	MIC ranges	MIC_50_	MIC_90_	% resistant	MIC ranges	MIC_50_	MIC_90_	% resistant
*Klebsiella pneumoniae*								
Amikacin (16)	3->256	>256	>256	94	>256	>256	>256	100
Cefoxitin (8)	64->256	>256	>256	100	>256	>256	>256	100
Ciprofloxacin (1)	>32	>32	>32	100	>32	>32	>32	100
Colistin (2)	1–16	1	8	44	0.5–8	1	3	17
Ertapenem (0.5)	>32	>32	>32	100	>32	>32	>32	100
Imipenem (1)	>32	>32	>32	100	4->32	>32	>32	100
Meropenem (1)	>32	>32	>32	100	>32	>32	>32	100
Tigecycline (2)	1->256	2	8	75	2–16	8	16	83
Escherichia coli*								
Amikacin (16)	**-**	-	-	-	1.5->256	24	>256	56
Cefoxitin (8)	**-**	-	-	-	12->256	>256	>256	100
Ciprofloxacin (1)	**-**	-	-	-	>32	>32	>32	100
Colistin (2)	**-**	-	-	-	0.5–12	1	2	11
Ertapenem (0.5)	**-**	-	-	-	>32	>32	>32	100
Imipenem (1)	**-**	-	-	-	>32	>32	>32	100
Meropenem (1)	**-**	-	-	-	>32	>32	>32	100
Tigecycline (2)	**-**	-	-	-	0.25->256	12	>256	56

*Only one isolate was encountered in Adan Hospital and was resistant to all antibiotics; bp = breakpoints

Comparatively, resistance to tigecycline was 83%. By contrast, only 17% were resistant to colistin with MIC_50_ and MIC_90_ of 1 μg/ml and 3 μg/ml, respectively. All, but one, of the *E*. *coli* isolates were from Mubarak Al Kabeer Hospital and they were fully resistant to cefoxitin, ciprofloxacin, ertapenem, imipenem and meropenem with MIC_90_s of >256, >32, >32, >32 μg/ml, respectively. Resistance rates against amikacin and tigecycline were 56% and 56%, respectively. They were moderately resistant (11%) to colistin, with MIC_50_ and MIC_90_ of 1 μg/ml, and 2 μg/ml, respectively.

### Carbapenemase genes

**Fig 3:**
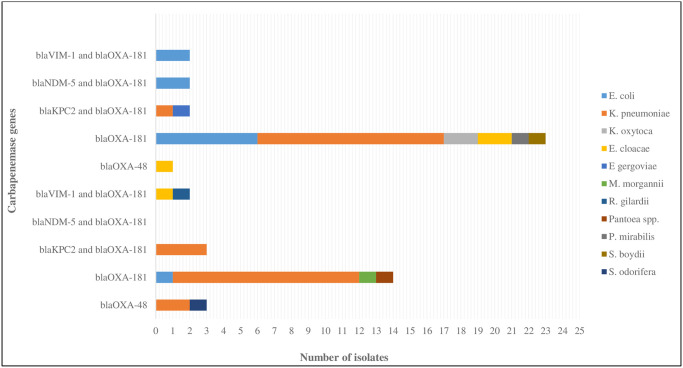
Prevalence of different carbapenemase genes harbored by CRE isolates from Adan and Mubarak Al Kabeer Hospitals.

A total of 56 (96.6%) out of the 58 CRE isolates harbored one or 2 carbapenemase-mediating genes as shown in Fig 3. Interestingly, 50 (89.3%) of the 56 carbapenemase-producing Enterobacteriaceae (CPE) isolates harbored *bla*_OXA-181_ gene either singly or in combination with other genes. Other genes detected were *bla*_VIM-1_ (4 isolates), *bla*_NDM-5_ (3), and *bla*_KPC-2_ (5). A total of 44 (78.6%) of the 56 isolates harbored single genes and 12 (21.4%) harbored 2 genes. As can be demonstrated in this Fig 3, 38 (86.4%) of those harboring single genes were positive for *bla*_OXA-181_, 5 (11.4%) *bla*_OXA-48_, and 1 (2.3%) *bla*_KPC-2_. By hospital, *bla*_OXA-181_ gene alone was harbored by 15 (60%) of 25, and 23 (69.7%) of 33 isolates from Adan and Mubarak Al Kabeer Hospitals, respectively. Of the 12 (21.4%) isolates that carried 2 genes, 4 (33.3%) were *K*. *pneumoniae* which harbored *bla*_KPC-2_, and *bla*_OXA-181_ (3 from Adan Hospital and 1 from Mubarak Al Kabeer Hospital). Two *E*. *coli* from Mubarak Al Kabeer Hospital and 1 *Salmonella* spp. from Adan Hospital carried both *bla*_NDM-5_, and *bla*_OXA-181_ genes. One each of *E*. *cloacae* and *Roseomonas gilardii*, and 2 *E*. *coli* harbored both *bla*_VIM-1_, and *bla*_OXA-181_ and one *E*. *gergoviae* carried *bla*_KPC-2_, and *bla*_OXA-181_.

The DNA sequences of *bla*_NDM_-_5_ and *bla*_OXA-181_ have been submitted to the GenBank database (US National Library of Medicine, National Center for Biotechnology Information, NCBI) under the accession numbers MK256964.1 (NDM) and MF774789.1 (OXA).

### Plasmid study

Analysis of plasmid content revealed that the 3 *K*. *pneumoniae* and 2 *E*. *coli* isolates contained multiple small and large plasmids, ranging between 80-200kb. Southern hybridization studies using *bla*_OXA-181_ probe localized the *bla*_OXA-181_ gene on the large 200kb size plasmid in the 3 *K*. *pneumoniae* isolates but the *bla*_NDM-5_ was located on the small 80kb size plasmid in the 2 *E*. *coli* isolates.

### Clonal relatedness of isolates

**Fig 4:**
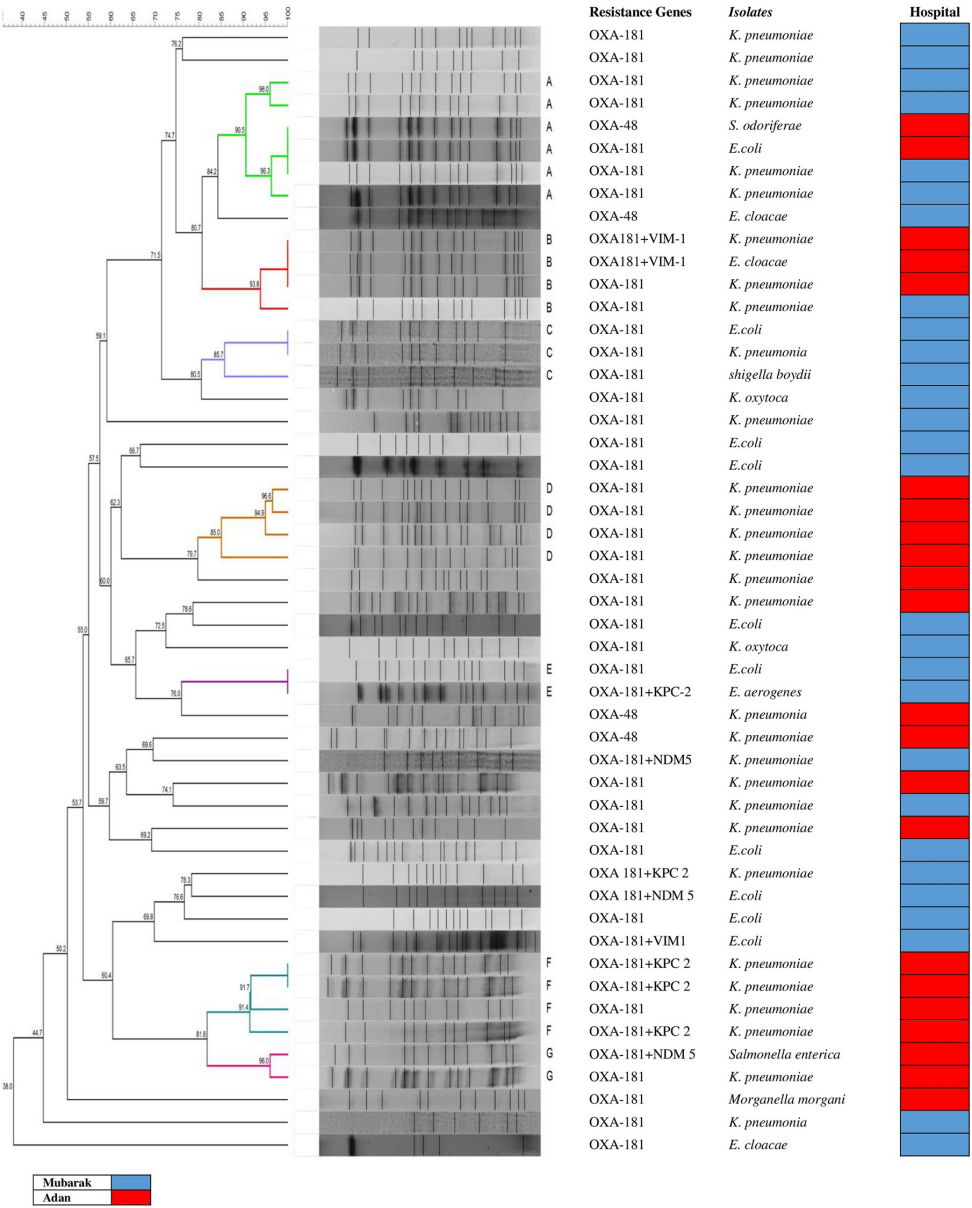
Dendrogram based on UPGMA cluster analysis of Dice similarity values of PFGE fingerprint patterns of 50 carbapenemase-producing Enterobacteriaceae (CPE) isolates in Kuwait. Band profiles of each strain are shown corresponding with the lines of the dendrogram. Seven pulsotypes (designated A, B, C, D, E, F, and G) diverging 85% criterion. The gene, isolate identification, and hospital are represented in the columns.

Clonal relationships of 50 carbapenemase-producing Enterobacteriaceae (CPE) isolates that carried either *bla*_OXA-181_ or *bla*_OXA-48_ genes from the 2 ICUs were determined by PFGE and demonstrated in Fig 4. The *Xba*I PFGE profile-based dendrograms showed that the overall level of similarity was 38%. However, the 85% criterion resolved 7 pulsotypes (designated A, B, C, D, E, F, and G). There was no single predominant pulsotype.

Further investigation revealed that certain isolates were collected from certain areas or units in the ICU e.g. in Mubarak Al Kabeer Hospital ICU, there were 7 subpulsotypes i.e. A1, A2, A5, A6, C1, C2, and E1 which were collected from unit D in the Medical ICU. While in Adan Hospital ICU, there were 13 different subpulsotypes i.e. A3, A4, B1, B3, D1, D2, D3, D4, F1, F2, F3, G1, and G2 which were isolated from the both Surgical and Medical ICUs.

## Discussion and conclusion

The prevalence of rectal colonization by CRE among patients has been described previously with rates varying from 0.5 to 12.2% in different areas of the world [26–31]. To our knowledge, this is the first study to report the prevalence of CRE gut colonization among ICU patients in Kuwait and, indeed, in the entire Gulf Cooperation Council (GCC) countries. We take cognizance of the fact that all colonized patients with CRE cannot be detected by depending on cultures collected according to the clinical condition of the patients. Detection of asymptomatic carriers in patients is exceedingly important because these patients may act as reservoir for transmission of CRE [15]. The main reservoir for CRE is the gut and fecal samples are usually used for its screening. But the isolation of CRE from stool specimens is often difficult due to the fact that these strains compose a small proportion of the gut flora and absence of efficient or standardized screening method for their detection makes the process more difficult [32, 33]. In addition, it has been shown that surveillance of low level resistance, such as IMP and OXA-48 producers, is difficult and, more often than not, the numbers are underestimated [34, 35]. Experience has shown that colonized patients with CRE are often transferred between wards within the same hospital or between different hospitals.

In this study, the overall prevalence of CRE among the 2 ICU patients was 9.8% which is lower than those of 12.2% reported in long-term care hospitals and acute-care hospitals in Japan [29] and 37.9% in Iran [36]. Similarly, higher figures of 12% have been reported in post-acute-care facilities wards in Israel [26] and 20.4% in the hematology-oncology wards in India [37]. In contract, our prevalence rate is higher than the reported figures of 6.6% in China [28] and 5.4% in USA although these studies were carried out in the ICU, medical, surgical, and acute rehabilitation units [27]. It is pertinent to point out that a direct comparison with these studies should be interpreted with caution as the settings and patients were different from ours. However, in studies, similar to ours, reported on hospitalized patients in Spain and France, the prevalence rates were 2.9% and 5.3%, respectively [38, 39]. Similarly, a prevalence of 3.8%, much lower than ours, was also reported in a large refractory nosocomial outbreak of KPC-producing *E*. *coli* in the Central Manchester University Hospital, UK [31] as well as in another study conducted in a large teaching hospital in Northern Italy, where the percentage of CRE rectal colonization dropped significantly from 0.2–0.05% after introduction of infection control interventions over a period of 30 months [30]. It is important to note that only a few of these studies were conducted on ICU patients. In concordance with the low community-acquired colonization rates encountered in our study, a study in Spain did report a much lower community-acquired colonization of rate of 0.4% [38].

An interesting characteristic feature found in our study was the preponderance of colonized Kuwaitis over non-Kuwaitis in each hospital, a finding that attained statistically significant difference (*P*<0.0001). This paradox has an important epidemiological implication because the population of Kuwait consists of about 35% indigenes versus 65% non-indigenes with strict segregation and yet more colonized patients were found among the Kuwaitis. Furthermore, the majority of these Kuwaitis had not traveled outside the country in the preceding year. It is conceivable that the CRE were present in the community and hospital environment in low numbers perhaps acquired silently from their foreign helpers who might have brought them from their respective countries over time.

The commonest CRE isolates in our study was *K*. *pneumoniae* followed by *E*. *coli* as in previous reports [37, 38] but a study from Japan had previously reported a reversal in prominence between the two bacteria [29]. It is noteworthy that 2 of the CRE colonized patients were asymptomatically positive for *Salmonella enterica* and *S*. *boydii*, a finding that has important infection control implications, especially in the ICU setting.

Not surprisingly, all our CRE isolates were resistant to the carbapenems, cephalosporins, and ciprofloxacin. This is concordant with the previous reports from Kuwait [6, 7]. Resistance to colistin and tigecycline appears to be on the ascendancy when compared to previous experience in Kuwait [8]. This is a dangerous trend as these antibiotics are the drugs of last resort for treating patients infected by multi-drug resistant (MDR) Gram-negative bacteria. The activities of the antibiotics tested against *E*. *coli* were equally poor although there were some differences in the susceptibility levels to carbapenems, aminoglycoside, colistin, and tigecycline. We foresee a situation where treatment options will be severely restricted in our hospitals should our patients become infected with these CRE strains.

By far the most important highlight of this study is the high prevalence (67.9%) of the CPE mediated by *bla*_OXA-181_ gene. This finding is new to Kuwait and very uncommon in the surrounding Gulf countries. Evidence from this study showed that this finding may represent rapid and widespread dissemination of *bla*_OXA-181_ in our country. Our assertion is based on the fact that most cases of infections and colonization have been in areas outside this region usually in patients who had recently traveled to Southeast Asia and the Asian Pacific region as these are the areas suspected to be the origin of this gene and an important reservoir of this carbapenemase gene. Although OXA-181-producing Enterobacteriaceae have also been reported in several other non-Asian countries [13], only a few anecdotal reports of clinical cases in Abu Dhabi, Oman and Lebanon have been reported from other countries in the Middle East [25, 40, 41]. It is pertinent to recognize that about 68% of the patients colonized in this study were indigenous Kuwaitis who had no history of previous travels to these regions. More intriguing is the fact that the 17% who were Indians did not admit traveling home in the last one year preceding admission to the ICU. Our speculation, at this time, is that the gene might have been imported by the returning expatriate Asian workers who form a very large population of domestic and middle class work force in the country. It is conceivable that this gene is present at low levels within this community and has found its way into the indigenous population.

Other interesting findings include the 2 *E*. *coli* and 1 *S*. *boydii* isolates that harbored *bla*_NDM-5_ and *bla*_OXA-181_. This is the first of such findings in Kuwait. The first report of a combination of these two genes was previously encountered in a patient, with urinary tract infection managed at a Singapore hospital in 2013, who had been transferred from Bangladesh [11]. Similarly, OXA-181 and NDM-5-producing CRE was reported in 2015 in a *K*. *pneumoniae* isolate from a South Korean patient transferred from a tertiary hospital in Abu Dhabi, UAE [42]. Recently, a cluster of NDM-5 and chromosomally mediated OXA-181-producing pan-resistant *K*. *pneumoniae* from 4 patients in Abu Dhabi with possible connections to the strains subsequently isolated in South Korea, was reported by Sonnevend *et al*., 2017 [25]. However, finding this combination in *E*. *coli*, like ours, has only been reported once before in Egypt [43] and Saudi Arabia [44]. It should be noted that in our study this combination was also found in one *S*. *boydii* isolated from a patient with diabetes mellitus and ischemic heart disease in Adan hospital representing the first of such report in this bacterium particularly in this country and the region. Furthermore, *bla*_OXA-181_ gene in association with other carbapenemase genes e.g. *bla*_NDM-1_ and *bla*_VIM-1_ has been reported in *K*. *pneumoniae* [42]. But the occurrence of this co-existence in *E*. *coli*, *Enterobacter cloaceae* and *Roseomonas gilardii* is alarming because of the belief that worldwide spread of this enzyme is a mirror image of that of NDM-1 [13].

As a result of the widespread colonization by OXA-181-producing CRE the isolates in the 2 hospital ICUs were investigated for any evidence of horizontal intra or inter–hospital spread using the PFGE. A relatively high percentage of the isolates (38%) were related. This suggests some form of spread within the ICUs of each hospital. Two isolates from both hospitals were also similar, a finding that can be explained, in part, by the occasional movements of patients between hospitals in Kuwait with possible transmission between the 2 hospital ICUs that remains a cause for grave concern in Kuwait. There is a significant correlation of isolates with identical pulsotypes between the date of collection and the hospital.

In our study, the gene encoding OXA-181 was carried on 200kb plasmid which is similar to the previous study reported by Castanheira *et al*. where *bla*_OXA-181_ gene was carried on plasmids of 200-250kb molecular sizes [45] but unlike the study of Potron *et al* which showed that the *bla*_OXA-181_ was located on a 7.6-kb ColE-type plasmid in *K*. *pneumoniae* isolate [46]. The NDM-5 plasmid, in our study, was located on a small 80kb plasmid which is smaller (110kb) than that detected in *E*. *coli* in Poland [47].

Limitations of this study include the following: only two centers were included and plasmid studies were limited to a few isolates. In addition, we did not study all carbapenemase resistance genes and other mechanisms of resistance especially for those 2 isolates which were carbapenem-resistant but carbapenemase genes negative.

In conclusion, the prevalence of rectal colonization by CRE in these 2 ICUs was higher than expected. Detection of *bla*_OXA-181_ and *bla*_NDM-5_ is new to the milieu of genes so far described in isolates from Kuwait. The emergence of *bla*_OXA-181_-mediated CRE is a cause for concern as there is the possibility of rapid horizontal spread among hospital patients. It is highly recommended that active surveillance cultures, strict patients isolation, and improved epidemiologic and hygienic situation should be carried out in our ICUs even when CRE prevalence remains relatively low in order to avoid an endemic trend.

## Supporting information

S1 Raw image (TIF)
