# Supplementary figures and images for: Prevalence of carbapenem-resistant Enterobacteriaceae and emergence of high rectal colonization rates of blaOXA-181-positive isolates in patients admitted to two major hospital intensive care units in Kuwait
Source: PLoS One. 2020 Nov 17;15(11):e0241971. doi: 10.1371/journal.pone.0241971 (PMC7671514; doi:10.1371/journal.pone.0241971)

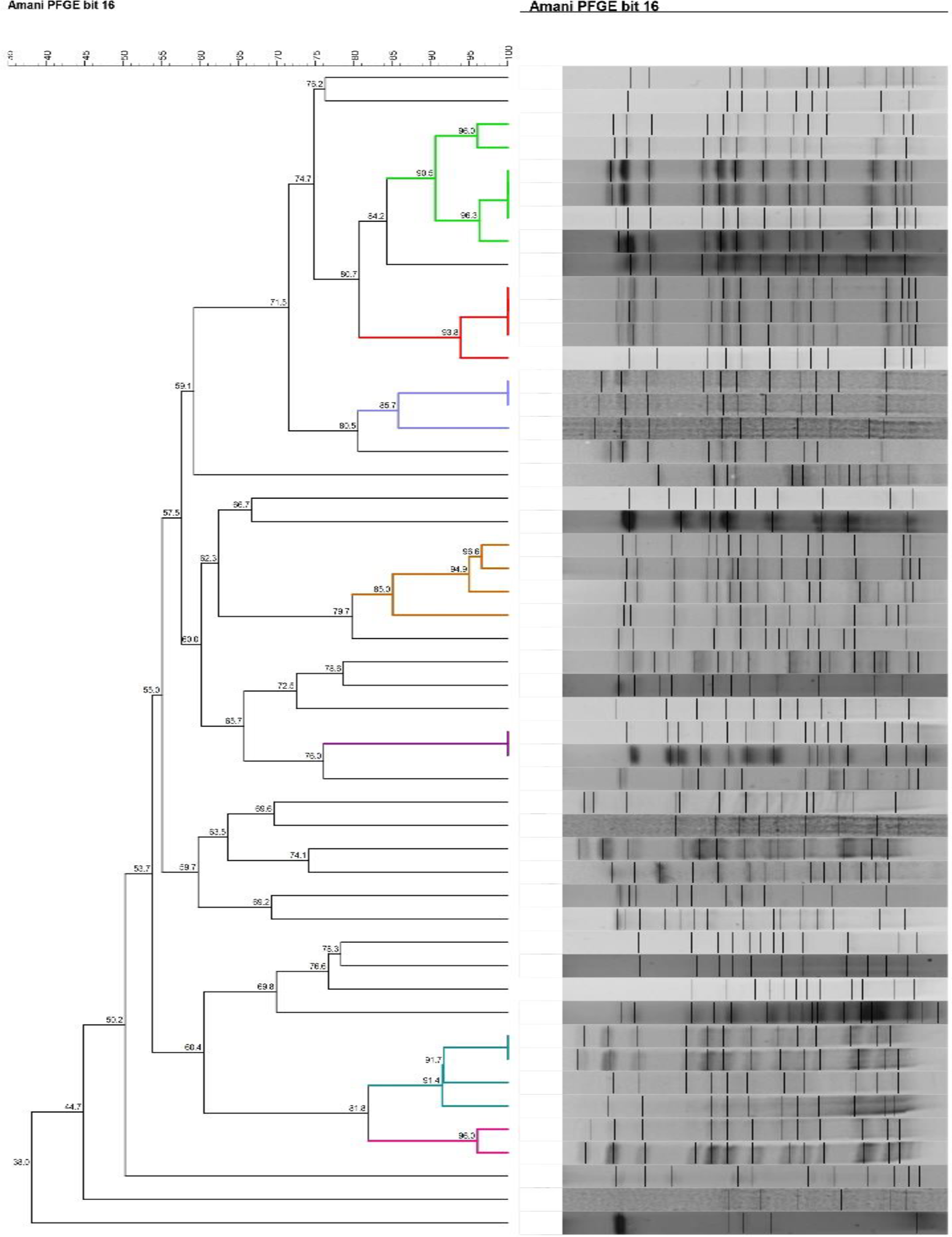

Supplement: S1 Raw image — (TIF) [file pone.0241971.s001.tif]
